# Measuring dominance certainty and assessing its impact on individual and societal health in a nonhuman primate model: a network approach

**DOI:** 10.1098/rstb.2020.0438

**Published:** 2022-02-28

**Authors:** Brenda McCowan, Jessica Vandeleest, Krishna Balasubramaniam, Fushing Hsieh, Amy Nathman, Brianne Beisner

**Affiliations:** ^1^ Department of Population Health and Reproduction, School of Veterinary Medicine, University of California, Davis, CA 95616, USA; ^2^ California National Primate Research Center, University of California, Davis, CA 95616, USA; ^3^ Department of Statistics, University of California, Davis, CA 95616, USA; ^4^ Colony Management Department, Yerkes National Primate Research Center Field Station, Lawrenceville, GA, USA

**Keywords:** hierarchy, dominance rank, network analysis, dominance certainty, individual health, societal health

## Abstract

The notion of dominance is ubiquitous across the animal kingdom, wherein some species/groups such relationships are strictly hierarchical and others are not. Modern approaches for measuring dominance have emerged in recent years taking advantage of increased computational power. One such technique, named Percolation and Conductance (Perc), uses both direct and indirect information about the flow of dominance relationships to generate hierarchical rank order that makes no assumptions about the linearity of these relationships. It also provides a new metric, known as ‘dominance certainty’, which is a complimentary measure to dominance rank that assesses the degree of ambiguity of rank relationships at the individual, dyadic and group levels. In this focused review, we will (i) describe how Perc measures dominance rank while accounting for both nonlinear hierarchical structure as well as sparsity in data—here we also provide a metric of dominance certainty estimated by Perc, which can be used to compliment the information dominance rank supplies; (ii) summarize a series of studies by our research team reflecting the importance of ‘dominance certainty’ on individual and societal health in large captive rhesus macaque breeding groups; and (iii) provide some concluding remarks and suggestions for future directions for dominance hierarchy research.

This article is part of the theme issue ‘The centennial of the pecking order: current state and future prospects for the study of dominance hierarchies’.

## 1. Introduction

The concept of dominance is ubiquitous across the animal kingdom including humans, wherein some species and groups such relationships are strict and others are more relaxed [1]. This phenomenon is particularly true in nonhuman primates, and even more specifically in the macaque genus (*Macaca* spp.) in which ‘dominance style’ has been viewed on a scale ranging from despotic to tolerant [2,3]. The notion behind the range of ‘strict’ to ‘relaxed’ is the degree to which a hierarchy is strictly linearly arranged, as in the classic seminal paper by Schjelderup-Ebbe [4] in describing pecking orders of chickens. Since then, a multitude of papers has been published examining and re-examining what ‘dominance’ actually means, for example, whether it is an individual attribute or a relationship and how it is defined either by aggression and/or submission, both topics of which have been considerably debated over decades of research [1,5]. Nevertheless, the concept of dominance has contributed greatly to our understanding of social structure in both human and group-living nonhuman animals [1]. Therefore, continuing to develop empirical approaches that capture more nuanced, biologically meaningful facets of dominance (e.g. rank, but also other facets: see below) remains an important, even critical endeavour for behavioural biologists.

Because dominance hierarchies and hierarchical structure are extremely prevalent in both human and animal societies [5], an accurate assessment of dominance structure is an important aspect of animal behaviour research. Functionally, dominance, and its metric usually represented as rank, is about *establishing, maintaining (and sometimes testing) predictability in relationships* at the group, individual and dyadic levels [6]. At a group level, the emergence of hierarchical structure in animal societies is important for understanding what selective pressures and processes have led to the appearance of different types of social order. At the individual level, social dominance has been linked with differential resource acquisition, individual health outcomes and fitness indicators in both humans and a wide range of group-living animal taxa [7–12]. Across social taxa, there is consistent evidence for high dominance rank to be associated with benefits such as increased access to food resources and mating opportunities, reproductive success and offspring survival and strong ties of social support through animals' affiliative and cooperative social interactions (reviewed in [13]). In some species or socio-ecological contexts, high-ranked individuals also experience less psychosocial stress, improved cardiovascular health, better immune function and lower levels of glucocorticoids than low-ranked individuals [14–18], a pattern also observed in most human populations [10]. On the other hand, being high-ranking also brings potential costs, such as higher parasite loads, and increased allostatic load via chronic stress levels in some species and/or socio-ecological contexts [9,19,20].

As there are multiple definitions for dominance, there are also many different methods for quantifying hierarchical structure [21–25]. Indeed, discrepancies in the estimation of dominance rank that may arise from using different methods likely impact its relationship to animal health and fitness, and how such effects are interpreted. The two primary classes of methods for quantifying dominance rank (one that calculates individual's rank via overall wins/success, and one that relies upon permuting a matrix of wins and losses) both yield a linear rank order for all individuals in a society. Thus, societies with nonlinear dominance structure (see [26] for brief review) require methods that can quantitatively represent that nonlinear structure in order for its effects on animal health and fitness to be properly studied.

The relative certainty, or ambiguity, of rank is another aspect of dominance that is not often examined, in part due to methodological limitations. In simple terms, the term ‘certainty’ refers to the likelihood an individual's rank might remain stable, i.e. unchanged through time, on account of the consistency in the outcome (wins and/or losses) of its agonistic encounters with its conspecifics. A few early studies on nonhuman primates introduced and used the term ‘rank stability’ quantified simply by changes to animals' rank across time, and further revealed that rank *in*stability was associated with negative health outcomes. For instance, Sapolsky [12] showed that rank *in*stability, as measured by rank changes can have an impact on individual health outcomes. Similarly, Shively & Clarkson [18] showed that among female long-tailed macaques that were forced into rank shifts via experimentally changing membership in small indoor-housed groups, individuals that changed (either lost or gained) ranks also had worsened coronary artery atherosclerosis. Together, these among other studies ([13] for review) show that the relative stability of one's dominance rank has health and fitness consequences for the individual.

But what if the concept of rank (in)stability needs to be extended further—to ask about the predictability or *certainty* of one's rank, or a specific dyad's rank relationship? The certainty of rank is essentially the *probability* or *likelihood* of a future rank change, given the current rank. And this is true as well as the potential negative (health) consequences even if ranks do not actually change. Therefore, analysing rank changes and rank certainty in tandem might help reveal the extent to which it is the actual change in rank that influences individual outcomes or the uncertainty associated with rank changes that have these effects. Yet because detecting rank certainty through its stability across time may not be feasible in the absence of temporal data on animals' wins and losses in agonistic interactions (thereby waiting to observe actual changes in rank), we require other methods to examine the impact of rank uncertainty without necessarily observed rank changes [27].

Complicating matters, in many animal taxa, individuals may interact infrequently either because they choose to do so or on account of socio-demographic or ecological barriers that they may face. Such barriers might also reduce the observability of animals, negatively impacting sampling effort [28]. Sparse and missing interactions are a common problem in behavioural field studies reliant on observational data and may reflect a true lack of interaction (e.g. intentional avoidance, and perhaps uncertain rank relationship) or sampling limitations. The study of rank uncertainty, beyond observed changes in rank, is therefore hampered by missing data from non-interacting dyads. Methods such as Percolation and Conductance (Perc) offer a way to properly characterize non-interacting dyads and therefore have the potential to explicitly examine rank uncertainty. Sparse and missing data also influence the accuracy of dominance calculations for some methods [28,29]. For instance, under the I&SI method, rank orders become inconsistent when 23–38% of the relationships are unknown [28]. When the linearity assumption is not met, assigning cardinal indices (e.g. David's Score, Elo-rating) can produce inaccurate and nonintuitive rank orders [30] thereby misrepresenting the actual dominance structure of a society. Further, missing data can also impact estimations of hierarchy linearity—zeroes in the win/loss matrix might reflect unsettled dominance relationships [31], which further suggests the potential for rank tiers (i.e. two or more animals occupy the same rank) or other nonlinear rank structure. Most methods assume linearity (and produce outputs that reflect this assumption), although the dominance hierarchy in many societies does not meet the criteria of linearity [26]. Rather than forcing a nonlinear dataset into a linear structure, methods are needed that can accurately represent such nonlinear structure. Two recently developed methods designed to handle nonlinear structure that also address sparsity in data are Perc [32,33] and ADAGIO [26].

In this focused review, we will concentrate on the Perc method, a network-based algorithm developed by members of our research team to assess dominance by evaluating information flow through the network [32–34]. In doing so, we will highlight the unique advantages of this method. First, we describe how Perc nonlinearly measures pairwise dominance relationships, even for non-interacting dyads, and provides a quantitative metric of ‘dominance certainty’ at both the level of the dyadic relationship and the individual (and can be extended to the level of the group), which provides valuable complimentary information to dominance rank. Second, we will summarize a series of published studies by our research team that illustrate the biological importance of ‘dominance certainty’, which can modulate or sometimes possibly even overshadow the importance of actual dominance rank, with respect to individual and societal health in captive populations of large rhesus macaque breeding groups. Lastly, we will provide some concluding remarks and suggestions for future directions for dominance hierarchy research.

## 2. Methodological considerations

### (a) Percolation and conductance

Percolation and conductance (referred to here as Perc, the name of the R package), first described in Fushing *et al*. [33] with algorithmic improvements presented in Fujii *et al*. [32], uses first direct and then indirect network connections (via paths of different lengths in the dominance network) to gather dominance information about all pairs of animals, particularly among non-interacting pairs. This network information is used to increase the volume of data available to estimate the probability that *i* dominates *j*. Briefly, a dominance network is constructed from a dataset of pairwise interactions, and all dominance paths are found between each pair of individuals. Then, information from indirect paths is treated as imputed or fractional ‘wins’; the contribution of paths is weighted based on network-wide directed transitivity (the proportion of triangles with a transitive pattern of links). When the higher order structure is transitive, indirect paths likely contain hierarchically structured information and thus are given their due weight. Conversely, when transitivity is low (e.g. when a network has a high degree of circular pathways), paths contain little dominance information and are not given much weight. Both direct wins and imputed ‘wins’ are then used to compute a probability that subject *i* outranks subject *j*. This matrix of dominance probabilities is reordered to maximize the probability of winning in the upper triangle, like other methods such as I&SI.

**Figure 1.**
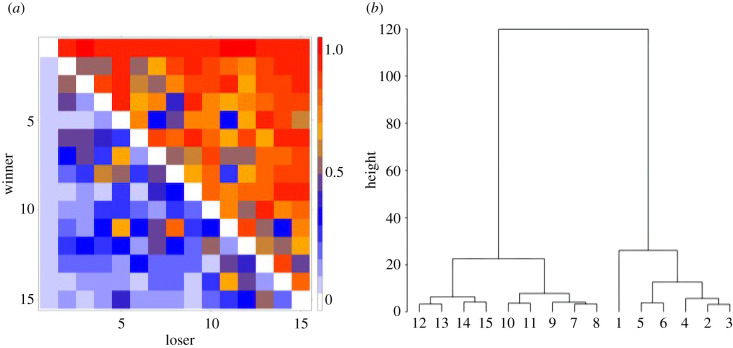
(*a*) Heatmaps of dominance probabilities (indicated by cell colour) with 0 indicating certain subordination of the row animal to the column animal, 1 indicating certain dominance of the row animal to the column animal and 0.5 indicating ambiguity in the relationship. Axis values represent individual dominance rank with 1 indicating the top ranked animal. Note that there is greater ambiguity in the centre of the hierarchy and along the diagonal. (*b*) Hierarchical clustering tree of rank distances (see electronic supplementary material for calculation methods). Animals closer to each other in the tree have more ambiguous relationships than those farther apart. This tree can be cut to identify rank tiers in which animals within each tier have more ambiguous dominance relationships with clearer dominance between tiers.

Perc makes no assumption of linearity. Although the ordering of the matrix in Perc can be taken as a linear rank order, this is by no means a necessary endpoint. The ordered dominance probability matrix can be examined further for evidence of nonlinear structure. Pairs of animals with indistinguishable ranks are visually apparent through generating a heatmap of the dominance probability matrix as cells (or clusters of cells) along the diagonal with values close to 0.5 (e.g. DP < 0.70). Further, two or more animals may be formally assigned the same rank (i.e. placed on the same rank tier) by generating a hierarchical tree of the distances between animals in the hierarchy and cutting the tree to produce clusters of individuals with similar or indistinguishable rank (figure 1). It is here that Perc offers an intriguing and important metric, known as ‘dominance certainty’.

### (b) Dominance certainty

**Figure 2.**
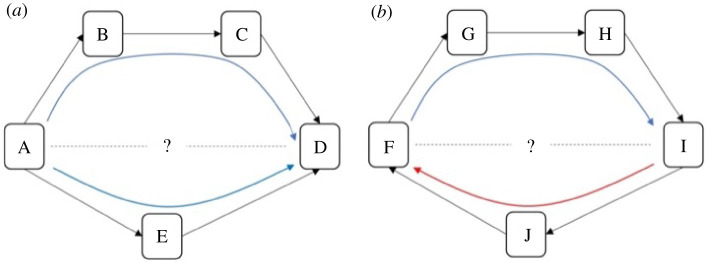
Inference of dominance rank and certainty using a network. (*a*) Although animals A and D do not directly interact, it can be inferred that A outranks D through the indirect pathways in the network. Certainty for this inference is increased when multiple pathways flow in the same direction (i.e. from A to D). (*b*) Although animals F and I do not interact, it can be inferred through the most direct pathway (through individual J) that I outranks F. Certainty for this inference, however, is lower due to the contradictory flow of dominance from F to I (through individuals G and H). Reprinted with permission from *PeerJ*, Vandeleest *et al*. [35]. (Online version in colour.)

**Figure 3.**
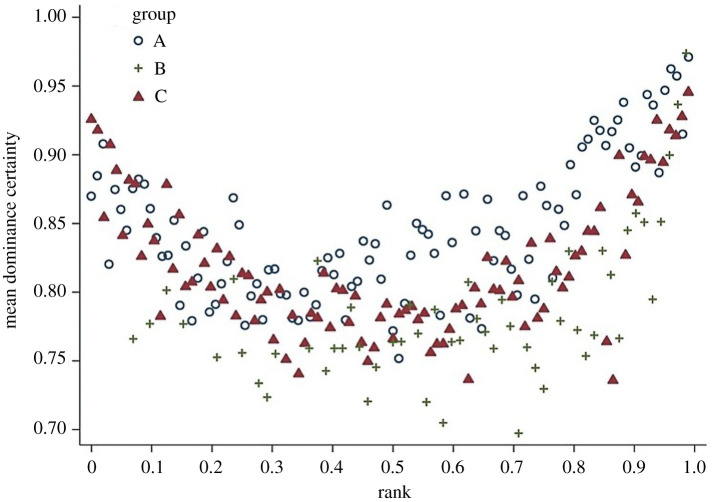
Rank and dominance certainty. Scatter plot of dominance rank and dominance certainty. Markers indicate group membership. Reprinted with permission from *PeerJ*, Vandeleest *et al*. [35]. (Online version in colour.)

As indicated above, by the use of information flow through a network, Perc provides two individual-level disentangled metrics relating to the dominance hierarchy: dominance rank (linearly ordered or rank tiered status relative to other animals in the group) and dominance certainty (the degree to which status is decided) [32,33]. This method characterizes the flow of status, that is the overall direction of aggression and submission, through pathways in the network and each individual's fit within that hierarchical flow to quantify both dominance rank and dominance certainty. Similar to most methods for measuring dominance rank, Perc uses direct aggression and/or submission data to create a dominance hierarchy. Unlike other methods, however, it also uses information from *indirect pathways* through the network of agonistic interactions to adjust the likely rank association between individuals and to measure the consistency of information flow through the network [33]. For example, in figure 2*a*, if animal A directs aggression at animal E (and E submits) and E directs aggression at D (and D submits), we can infer that A likely outranks D even if they have never been observed to interact. Greater consistency in the direction of dominance pathways from A to D (across multiple paths) results in higher certainty that A outranks D, whereas evidence of inconsistent direction (e.g. between F and I) reflects dominance ambiguity in figure 2*b*. This method thus solves the problem of sparse or missing data (up to a threshold dependent on the size of the interaction matrix and the proportion of zero dyads comprising the interaction matrix, e.g. very sparse datasets will always suffer from this problem) in the win/loss matrix described above by using these dominance pathways as additional sources of information about each pairwise dominance relationship. This method additionally provides *a measure of how well the direction of each individual's dominance interactions fit, on average, within the larger group-level pattern* of aggression from dominants to subordinates; this is the measure we call dominance certainty, which is calculated as the average level of certainty across all of an individual's pairwise relationships. This metric of dominance certainty, therefore, provides complimentary information to dominance rank, as seen in the U-shaped relationship between dominance rank and dominance certainty in figure 3. First, dominance certainty exhibits a nonlinear relationship with dominance rank, where high- and low-ranking animals appear to show greater certainty than middle-ranking animals. Second, however, we note that that individual variation in dominance certainty exists across individuals who are similar in rank, resulting in the possibility to look at the importance of dominance certainty, beyond or in interaction with rank, in relationship to both individual and group attributes. Notably, this metric of dominance certainty can reflect either inferential (statistical) certainty, biological certainty, or both. When transitivity of a dominance network is low, little information is gained from indirect pathways and, therefore, low certainty among non-interacting dyads is more likely due to lack of information. However, in cases where transitivity is high, indirect paths do provide important information and certainty among non-interacting dyads is more likely to be more reflective of biological uncertainty. Thus, if a system is sufficiently measured (or in other words if a group or population of animals is sufficiently sampled), certainty among non-interacting individuals reflects true (un)certainty in dominance and not just uncertainty due to lack of information. That is, non-interacting dyads are true, biologically meaningful zeros that represent animals that either actively choose to avoid interacting, and/or are hampered by socio-demographic or ecological barriers. Perc measures such transitivity and can compare the distribution of paths across interacting and non-interacting dyads to assess inferential versus biological (un)certainty (see electronic supplementary materials).

We now turn to the application of these methods in a series of published work by our and other research teams.

## 3. Applications

### (a) Dominance certainty as a predictor of individual health

**Figure 4.**
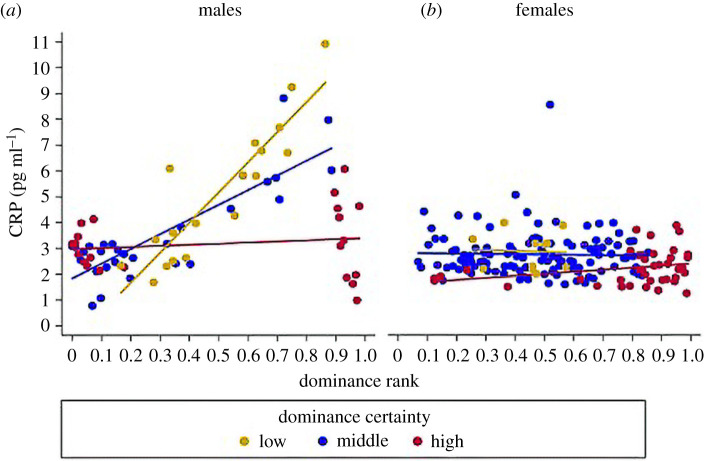
Interaction of status (as measured by percent outranked (1 being highest rank and 0 being lowest rank) and status certainty (from conflict data) on levels of C-reactive protein (CRP) in male and female rhesus macaques (range in dominance certainty similar for each sex). Reprinted with permission from *PeerJ*, Vandeleest *et al*. [35]. (Online version in colour.)

Indicators of social rank in humans, such as socioeconomic status, can be a source of stress. Yet studies on the presence and direction of this relationship between rank and stress in animal societies have been equivocal in terms of health impacts [9,36]. Studies from our research group on rhesus macaques show that certainty or ambiguity of one's dominance/status relationships may have a greater impact on health than actual rank itself. Using Perc [32,33], we assessed dominance certainty which again quantifies the number of ambiguous relationships per individual using both direct and indirect network connections. Importantly, dominance certainty in rhesus macaques relates to health outcomes in situations where rank does not. In Vandeleest *et al*. [35], analyses of both physiology and stress-related health measures demonstrated that ambiguous status relationships exhibit a dose-dependent relationship with greater incidence of diarrhoea and higher CRP, cytokine and hair cortisol levels, and for some outcomes this was dependent on sex (figure 4) [35,37]. Additionally, for the pro-inflammatory cytokines, dominance certainty modified the effect of rank. Male individuals with lower status certainty had poorer health only if they were also high-ranking. In other words, ambiguous status relationships only negatively impacted cytokine levels for those that stood to lose their high status (i.e. low-ranking, low-certainty individuals stand to *gain*). Females did not exhibit this relationship between rank and certainty on these health metrics, likely because rank is inherited via matriline membership and thus other aspects of their relationships (e.g. affiliation) may govern their health dynamics. Finally, other aspects of animals' social network connectedness may also interact with dominance certainty to influence their health, and specifically their susceptibility to infectious disease. Across two socially stable, captive groups of rhesus macaques, we found that possessing strong social connections within their affiliative grooming networks seemed to socially buffer individuals from the risk of infection from a bacterial pathogen, *Shigella* spp. Interestingly, we found that this ‘social buffering’ effect was particularly pronounced among macaques that had more ambiguous dominance relationships compared to those with greater certainty suggesting that those individuals with more ambiguous relationships disproportionally benefitted from this affiliative social buffering [38].

A powerful insight from the above summarized results is that the certainty (or predictability) about one's position in the network of status relationships may be just as important as absolute rank in predicting health outcomes. Further, this metric of individual-level certainty is based upon a global pattern of directional flow of interaction paths, illustrating the potential for multi-scale social structure (e.g. how one's interactions are embedded within the structure/geometry of their social community) to impact health. We, therefore, can quantify how much uncertainty or how many uncertain relationships, and with whom, are sufficient to impact health. These results from a nonhuman primate model clearly indicate that knowledge beyond direct relationships, and indeed beyond simple socioeconomic class designations, is critical to understanding health. These findings underscore that further exploring the complexity and multidimensionality of social relationships is important for understanding health impacts of social life [6,14,39,40].

### (b) Dominance certainty as predictors of societal health

**Figure 5.**
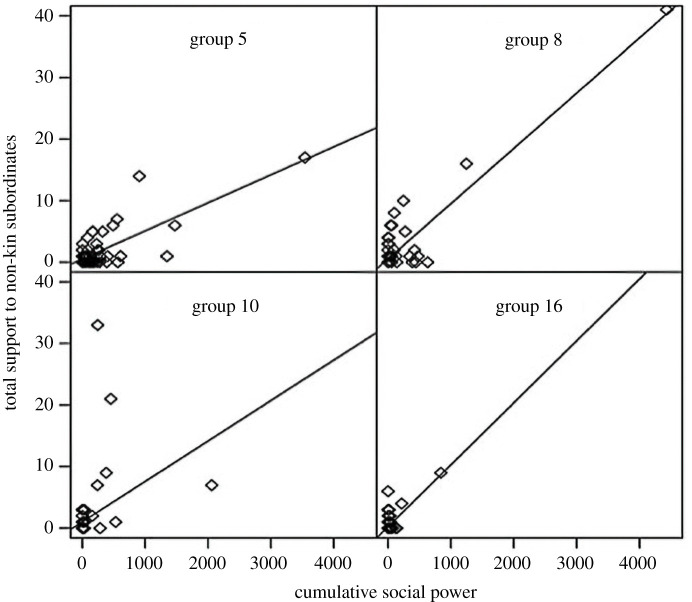
Frequency of support given to non-kin subordinates (i.e. policing) by cumulative social power. Reprinted with permission from *Am. J. Phys. Anthropol.*, Beisner *et al*. [42].

**Figure 6.**
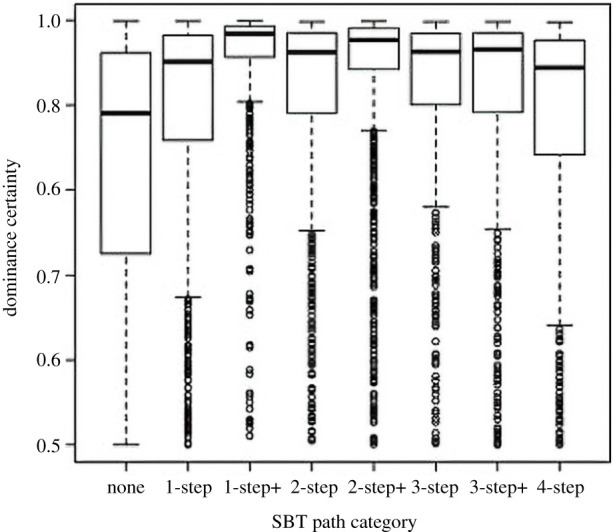
Dyadic dominance certainty plotted for all dyads with different lengths and combinations of peaceful silent-bared teeth (pSBT) signalling pathways. Boxes represent the interquartile range and the black bar is the median dominance. Reprinted with permission from *Am. J. Phys. Anthropol.*, Beisner *et al*. [42].

Beyond individual health, dominance certainty has also been tied to societal health in captive rhesus macaques in the form of social stability. In a variety of species, alpha males and/or high-ranking individuals ‘police’ conflicts among group members, a conflict management strategy that promotes social stability [41–44]. Individuals that police do so at great physical risk, and they are only likely to do so if such risk is substantially minimized. This risk is best reflected by the metric known as social power: receipt of frequent subordination signals from a diversity of group members yields a high degree of consensus over the social power of the receiver, who then polices more frequently and successfully (figure 5) because their risk of retaliation is low [41,42,44–46]. Networks of subordination signals, from which social power calculations are derived, exhibit important properties—the networks are acyclic (i.e. no circular paths exist), pathways of signals converge on a small number of conflict policers in the group, and these indirect signal pathways convey similar information to directly received subordination signals [42]. Dyadic dominance certainty is higher for pairs when an indirect path is present, compared to no paths at all or a direct signal only (figure 6), and calculations of social power using both direct and indirect subordination signal pathways (aka cumulative social power) are as good as, or better than, calculations using only direct signals received [42]. We argue that such status signalling networks are critical for communicating formally settled dominance relationships, thereby promoting social stability in complex and large primate systems. Furthermore, the relationship between dominance certainty and peaceful silent-bared teeth (display) signalling pathways highlights an important connection between two aspects of rhesus macaque society, that is aggression and status signalling [47], demonstrating an interdependence in dyadic aggression and status signalling as it relates to social instability. In a hierarchical system, the relationship between different networks can decouple if the power structure of the system disintegrates [48]. In rhesus macaque society, pSBT networks are representative of this power structure, and relate both to dominance certainty, which is important for relationship stability and, therefore, group stability, and also support the additional stabilizing mechanism of policing. We, therefore, argue that this network of formal dominance relationships and their certainty is central to rhesus macaque social stability [42].

### (c) Dominance certainty influences multiplex centrality

**Figure 7.**
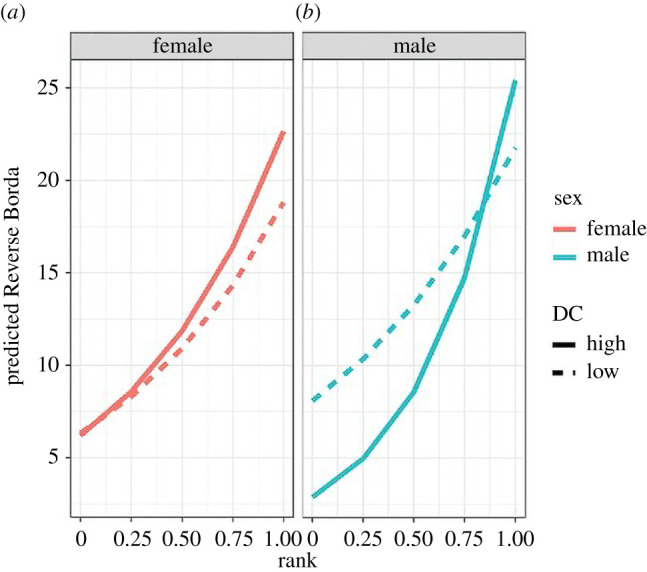
Relationship between Reverse Borda rank (a multiplex metric designed in Pósfai *et al*. [59]) and rank, sex and dominance certainty. Predicted Reverse Borda ranks for (*a*) females and (*b*) males, calculated at two levels of dominance certainty from the observed range of values: high dominance certainty (transformed DC = 0.1) and low dominance certainty (transformed DC = 0.3). Reprinted with permission from *PeerJ*, Beisner *et al*. [63]. (Online version in colour.)

In recent years, and as evidenced in the study described above, a major uptick in interest over the modelling of more complex multifaceted relationships has emerged, known generally as multilayer networks [49–53]. Researchers of social status in nonhuman animals are also recognizing with greater frequency that social status involves not just dominance relationships but also other relational interactions that include both affiliative and agonistic behaviours [10,54–57]. As such, techniques for more flexibly evaluating the importance of multiplex components to the understanding of relationships have emerged [58–62]. Using the technique developed by Pósfai *et al*. [59], we asked whether dominance certainty influences the role that individuals play in their multiplex networks [63]. Interactions among sex, dominance rank, and dominance certainty showed that among males, but not females, the effect of dominance rank on multiplex centrality (measured by consensus or Borda rank) depended upon the relative certainty or ambiguity of the male's dominance rank (figure 7). High dominance certainty was associated with lower multiplex centrality in low-ranked males whereas having low dominance certainty (i.e. greater ambiguity surrounding your fit in the hierarchy) was associated with higher multiplex centrality in low-ranked males. By contrast, among high-ranked males, high dominance certainty was associated with higher multiplex centrality (figure 7). These results suggest that certainty in dominance relationships influence the role males play in multiplex networks but such influence is dependent on one's relative rank. Similar to the inflammatory cytokines, this interaction between dominance rank and certainty in males, but not females, suggests that an evaluation of one's place and fit in the hierarchy can have a profound effect on one's role in a diverse but interconnected set of social contexts, which in turn likely has important effects on health [35,64,65].

### (d) Dominance certainty and the evolutionary origins of social structure

In Balasubramaniam *et al*. [66], we conducted a comparative study of aspects of macaque dominance and grooming social networks across 34 macaque groups representing 10 species of varying ‘social styles’. This analysis revealed that the average, group-level dominance certainty of macaques (the average of dominance certainties across individuals in a group) showed a strong phylogenetic signal, i.e. was more similar among more closely related compared to distantly related species [66]. This finding extended previous comparative studies that detected phylogenetic signals in global aspects of dominance hierarchies—specifically hierarchical steepness [31] and dominance asymmetry or counter-aggression [67]—that are based on dyadic win/loss encounters [68,69]. Specifically, the detection of phylogenetic signals in dominance certainty suggests that even more complex traits such as network-wide flow patterns of dominance information in these primates may have a strong ancestral component.

### (e) Other applications

Since its original publication, Perc has been used by a number of investigators to generate dominance ranks and to evaluate the importance of dominance certainty to a lesser extent. From 2016 to the present day, Perc has been cited in a total of 29 publications (a few are reviewed below). Among just studies focusing on nonhuman primates, studies implementing Perc have ranged from testing phylogenetic and socio-ecological frameworks of primate social structure [66]; to assessing the links between dominance certainty, health and (by extension) well-being of captive macaques [35,38]; to determining the effects of rank on the socio-ecology of behavioural flexibility and risk-taking behaviours by wild primates through their interacting with humans and other anthropogenic factors [70–72]. Outside of nonhuman primates, Perc has also been used to generate metrics of transitivity for studies of sociality in ducks [73]. Of these 29 publications, nine have explicitly calculated and used the metric of dominance certainty to address a diversity of questions [35,38,42,63,66,74–77]. The rest have used it to measure rank. Few studies to date have used Perc to examine nonlinearities in hierarchies, although as stated earlier this is one of its key features. Two of the most recent studies used dominance certainty to assess physiological stress at the individual level during experimental social instabilities. In one study, Linden *et al*. [77] found that lower dominance certainty was associated with greater levels of hair cortisol in individuals nine months after a new group formation in captive rhesus macaques. In the second study, Wooddell *et al*. [78] found that dominance certainty was lower in individuals undergoing experimental social instabilities but did not find an association with hair cortisol. Finally, Funkhouser *et al*. [79] used Perc, among several other ranking methods, in a comparative study in which they revealed context-dependent differences in dominance hierarchies and individuals' social roles across captive chimpanzees (*Pan troglodytes*) and wild Tibetan macaques (*Macaca thibetana*).

Importantly, aside from the above biologically focused empirical studies, Perc has also recently been used in quantitative, methodological advancements in the estimation of dominance ranks. Villette *et al.* [34] investigated the performance of seven alternative ranking methods (for a total of 13 metrics), including two metrics in Perc, by data splitting a 3-year dataset of aggressive interactions in vervet monkeys. Each method's performance was assessed by determining whether individual ranks/ratings/scores obtained from the training dataset could successfully predict the outcome of the aggressive interactions that occurred in the testing dataset. Perc outperformed 9 of the other 11 methods and exhibited reliability in such predictions when training datasets were both short and long in duration (time over which data were collected) [34].

Clearly, given its evident but currently limited scope in use, our hope is that this focused review of Perc's uses, and especially the quantitative metric of certainty it outputs, will encourage others to think about how they might use approaches like Perc to frame new questions that address the complexity of dominance relationships that go beyond more simplistic measures of linear rank and include metrics of dominance certainty.

## 4. Conclusion and future directions

Hierarchical dominance relationships clearly have and continue to play a critical role in multiple aspects of group-living animal societies. Functionally, dominance and rank are thought to establish, maintain and test predictability in social interactions, but we often do not measure the actual predictability of rank relationships. Historically we have been limited in asking these types of questions because of the constraints in our ability to sufficiently measure the complexity inherent to these relationships. These major constraints include issues with sparsity of data, a lack of metrics that capture nonlinearity in dominance and a need for quantitative metrics of certainty (uncertainty) or predictability in these relationships. New network-based approaches, such as Perc, are addressing these limitations which have allowed us to begin to ask new questions about the importance of certainty in dominance relationships before actual rank changes occur. These approaches have also revealed that certainty in dominance relationships can modulate how dominance rank is associated with important metrics of social structure as well as individual and societal health, underscoring that dominance certainty has not only inferential but also biological significance. As such, future studies should consider Perc or other similar types of approaches for assessing the inherent complexity of dominance relationships as an opportunity to revise the types of questions one can ask about dominance rank (and its underlying moderators such as certainty) in relationship to both social dynamics and individual health. Finally, we encourage investigators to continue to move beyond the concept of ‘rank’ as a *proxy* for individuals' roles in social groups toward a more direct and thus deeper understanding as to what such rank reflects, such as the *specific roles* these individuals play in their social networks (e.g. cohesion, coalitionary support, policing, etc.). In all, therefore, the concepts of dominance and hierarchy remain very useful, but continue to require targeted fine-tuning to address the complexity and interconnectedness of social relationships and their certainty, as well as their influence on not just individuals’ health but also the structural dynamics and robustness of their social networks.

## Data accessibility

The data that support the findings of this study are included in electronic supplementary material [80].
